# Molecular Characterization of Gram-Negative Bacilli Isolated from a Neonatal Intensive Care Unit and Phenotypic and Molecular Detection of ESBL and Carbapenemase

**DOI:** 10.3390/antibiotics14040342

**Published:** 2025-03-27

**Authors:** Thaís Alves Barbosa, Maria Regina Bentlin, Lígia Maria Suppo de Souza Rugolo, João César Lyra, Adriano Martison Ferreira, Ana Cláudia Moro Lima dos Santos, Nathalia Bibiana Teixeira, Letícia Calixto Medeiros Romero, Carlos Magno Castelo Branco Fortaleza, Maria de Lourdes Ribeiro de Souza da Cunha

**Affiliations:** 1 Department of Infectious Diseases, Botucatu Medical School, São Paulo State University (UNESP), Botucatu 18618-687, Brazil; thaalvesb@hotmail.com (T.A.B.); carlos.fortaleza@unesp.br (C.M.C.B.F.); 2 Department of Genetics, Microbiology and Immunology, Institute of Biosciences, São Paulo State University (UNESP), Botucatu 18618-691, Brazil; ana.moro@unesp.br (A.C.M.L.d.S.); nathalia.teixeira@unifsp.edu.br (N.B.T.); leticia.calixto@unesp.br (L.C.R.M.); 3 Department of Pediatrics, Botucatu Medical School, São Paulo State University (UNESP), Botucatu 18618-687, Brazil; regina.bentlin@unesp.br (M.R.B.); ligia.rugolo@unesp.br (L.M.S.d.S.R.); joao.lyra@unesp.br (J.C.L.); 4 Clinical Laboratory-Microbiology, Botucatu Medical School, São Paulo State University (UNESP), Botucatu 18618-687, Brazil; adriano.martison@unesp.br

**Keywords:** colonization, infection, extended-spectrum β-lactamases, biofilm

## Abstract

Introduction: The increase in the rates of multidrug-resistant bacteria in healthcare environments has been recognized as a global public health problem. In view of the scarcity of data on the neonatal population, this study aimed to provide information on the genotypic and epidemiological characteristics of Gram-negative microorganisms isolated from colonization and infection sites in neonates admitted to a tertiary university center of high complexity. Methods: Enterobacterales and non-fermenting Gram-negative bacilli previously collected in a prospective cohort study were submitted to genotypic identification, detection of extended-spectrum β-lactamases (ESBL), carbapenemases and biofilm production, detection of specific virulence markers in *Pseudomonas aeruginosa*, and typing by pulsed-field gel electrophoresis. Results: The data found here revealed higher rates of infection by *Klebsiella* spp. and *Serratia marcescens* that caused bloodstream infection and pneumonia, respectively. In this study, high biofilm production was observed, with 95.0% of Enterobacterales and 100% of non-fermenting Gram-negative bacilli being producers. Most of the *P. aeruginosa* isolates carried pathogenicity factors such as alginate, hemolytic phospholipase C, exotoxin A, and rhamnolipids. The phenotypic analysis of ESBL revealed that 16 (5.3%) isolates produced these enzymes. Four of these isolates (66.7%) carried the CTX-M-9 gene, three (50%) carried the TEM gene, and one (16.7%) was positive for the SHV and CMY-2 genes. Univariate and multivariate Cox regression analyses were used to identify risk factors for colonization and infection by Gram-negative microorganisms. The results of multivariate analysis revealed that biofilm production by these microorganisms was associated with the persistence of colonization by the same pathogen in the newborn and increased by 75% the daily probability of the newborn developing infection. The production of ESBL also increased the daily probability of infection by 46.8 times. Conclusions: Enterobacterales showed average biofilm production, while the majority of non-fermenting Gram-negative bacilli were strong producers. The present data increase our knowledge of the molecular epidemiology of important Enterobacterales species, with emphasis on ESBL-producing *Enterobacter cloacae* and *Klebsiella pneumoniae* with emerging epidemiological potential in the neonatal intensive care unit of a tertiary university hospital. Furthermore, the results highlight the need for the monitoring and implementation of control measures and for restricting the use of broad-spectrum antibiotics.

## 1. Introduction

Gram-negative bacilli are important pathogens associated with healthcare-related infections (HAIs) in neonatal intensive care units (NICUs) [1].

Extended-spectrum β-lactamases (ESBL) are enzymes that hydrolyze the beta-lactam ring [2] and are encoded by genes located on chromosomes or extrachromosomal elements such as plasmids or transposons [3,4]. The first ESBL was detected during a hospital outbreak of *Klebsiella pneumoniae* in 1982. Since then, more than 200 variants have been identified and have spread rapidly worldwide [5]. Outbreaks of ESBL-producing microorganisms in NICUs pose a threat to the health of newborns [6,7], increasing morbidity and mortality rates [8,9].

The biofilm production capacity of these microorganisms further complicates antimicrobial therapy [10].

Surface adherence is one of the prevailing modes for bacterial growth, while the biofilm environment supports the development of antimicrobial resistance [10,11].

Our understanding of how this lifestyle influences the evolution of antimicrobial resistance, whether mediated by different population genetic dynamics or molecular mechanisms, is limited. One example is that the close proximity of cells in biofilms may facilitate the horizontal transfer and persistence of resistance genes in bacterial populations [10,11,12].

Among Gram-negative microorganisms, *Pseudomonas aeruginosa* is one of the main etiological agents involved in HAIs. These infections are impossible to eradicate because of the resistance of this pathogen to various antimicrobials and the production of several virulence factors [13]. Biofilm production is considered the main virulence factor of *P. aeruginosa*; in addition, this microorganism produces phospholipase, alginate, exoenzyme S, cytotoxins, elastases, exotoxin A, and rhamnolipids as pathogenicity factors that exert different effects on the host’s immune system [13].

Molecular typing tools are essential to track these strains, contributing to the assessment of outbreaks and recurrent infections and to the understanding of clonal dissemination, facilitating epidemiological investigation [14,15]. Therefore, this study aimed to determine the mechanisms of resistance to antimicrobials by analyzing the production of β-lactamases and carbapenemases, biofilm production, specific virulence factors in *P. aeruginosa*, and prevalent clones.

## 2. Results

### 2.1. Strains

A total of 179 newborns admitted to the NICU of the University Hospital of the Botucatu Medical School (HFMB), SP, Brazil, were studied; 97 (54.2%) were colonized by Gram-negative microorganisms and six (3.3%) were infected by species of Gram-negative bacteria. During the study period, 600 positive colonization samples showing growth of microorganism were obtained. Of these, 350 exhibited specific morphology and staining for Gram-negative bacilli (Table 1 and Table 2).

### 2.2. Determination of Infection in Newborns

During follow-up, 34 (36.5%) newborns with infection were found throughout the hospitalization period. Of the 97 newborns colonized by Gram-negative microorganisms, 6 (6.2%) were confirmed by microbiological culture. Among Gram-negative pathogens isolated from clinical samples, there were two *K. pneumoniae* and one *Acinetobacter baumannii* isolated from blood culture, one *Proteus mirabilis* found in urine culture, and two *Serratia marcescens* and one *Klebsiella oxytoca* isolated from tracheal aspirate.

### 2.3. Biofilm Production

Biofilm production was assessed in 350 Gram-negative bacilli; of these, 335 (95.7%) were biofilm producers.

Biofilm production was classified as strong, medium, or weak according to the optical density obtained. Among Enterobacterales isolates, 285 (95%) were producers, including 22 (7.7%) strong producers, 81 (28.4%) medium producers, and 182 (63.8%) weak producers. In the group of non-fermenting Gram-negative bacilli (NFGNB), 50 (100%) isolates were producers, including 23 (46%) strong producers, 11 (22%) medium producers, and 16 (32%) weak biofilm producers.

*Enterobacter cloacae* was the species that showed the highest rate of biofilm production (96.3%) when compared to other Enterobacterales. In the group of NFGNB, 100% of the *P. aeruginosa* and *A. baumannii* isolates showed strong production.

### 2.4. Production of Extended-Spectrum β-Lactamases

The disc approximation test was performed for the 300 Enterobacterales isolates. Of these, 16 (5.3%) isolates demonstrated the appearance of the phantom zone and distortion of the halo around the β-lactam disc, indicating the production of ESBL. Six of the 16 isolates producing ESBL phenotypically harbored resistance genes; of these, four (66.7%) carried the CTX-M-9 gene, three (50%) carried the TEM gene, and one (16.7%) was positive for the SHV and CMY-2 genes (Table 3).

### 2.5. Production of Carbapenemases

The 350 Gram-negative microorganisms studied did not produce carbapenemases. When submitted to the initial screening tests, all isolates were susceptible to imipenem, ertapenem, and meropenem.

### 2.6. Virulence Profile of Pseudomonas aeruginosa Isolates

Genotypic determination of virulence markers was performed in 38 *P. aeruginosa* isolates. The evaluation showed that all 38 isolates (100%) were positive for the alginate D gene, 21 (55.3%) for the gene encoding exoenzyme S, 38 (100%) for the hemolytic phospholipase C gene, 34 (89.5%) for the gene encoding non-hemolytic phospholipase C, 38 (100%) for the exotoxin A gene, 38 (100%) for the alkaline protease gene, 38 (100%) for the elastase gene, and 38 (100%) for the gene encoding rhamnolipids.

### 2.7. Clonal Profile of the Isolates–Pulsed-Field Gel Electrophoresis

A total of 186 Enterobacterales were analyzed by PFGE. Of these, 70 (37.6%) corresponded to isolates of *E. cloacae*, 44 (23.6%) to *S. marcescens*, 33 (17.7%) to *Escherichia coli*, 14 (7.5%) to *Enterobacter aerogenes*, 11 (5.9%) to *K. oxytoca*, 5 (2.7%) to *K. pneumoniae*, 4 (2.1%) to *Citrobacter freundii*, 3 (1.6%) to *P. mirabilis*, and 2 (1.1%) to *Citrobacter diversus*. Among NFGNB, 16 were analyzed by PFGE, of these, 11 (68.7%) corresponded to isolates of from *P. aeruginosa* and 5 (31.3%) to *A. baumanni*.

**Figure 1:**
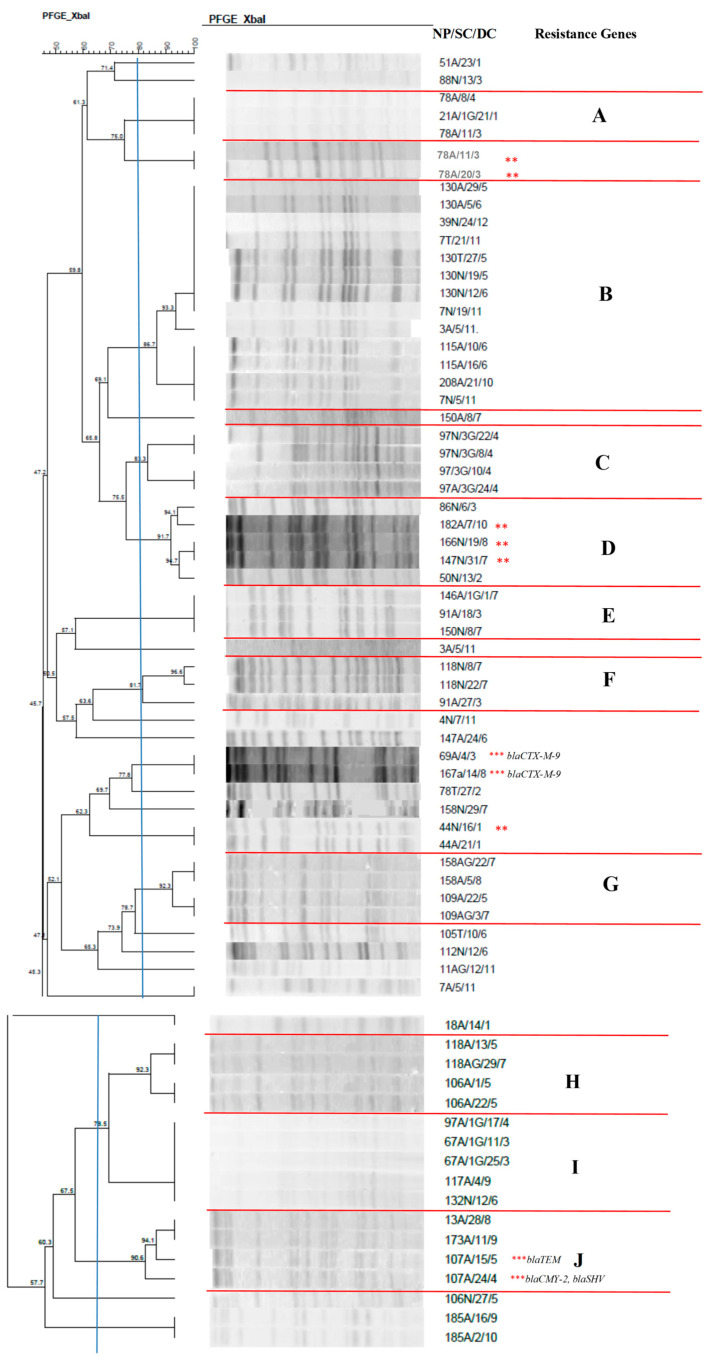
Dendrogram generated by Dice/UPGMA analysis (Bionumerics Applied Maths) [optimization: 1%, tolerance: 1.5%] of PFGE-XbaI profiles of *Enterobacter cloacae* isolates from newborns. Note: ** ESBL-producing isolates confirmed by the disc approximation technique; *** ESBL-producing isolates that carried resistance genes. NP: patient number; SC: site of collection; DC: date of collection (day, month); A: anal; T: tracheal. Letters A–J: Clusters, isolates with 80% or more similarity.

Analysis of the clonal profile of *E. cloacae* (Figure 1) revealed a polyclonal profile in most of the isolates, with differences between strains. There were 10 clusters showing similarity ≥80%, which were named A–J.

Cluster B, with 13 isolates, contained isolates with identical profiles or a high rate of similarity between different newborns, indicating possible transmission between them. The presence of isolates from the nasal, anal, and tracheal mucosa of the same neonate was also observed, demonstrating that the newborn is colonized by the same lineage at different sites. This fact can also be seen in cluster C.

Similar to the findings mentioned above, clusters A, D, E, F, G, H, I, and J contained identical isolates from different newborns.

**Figure 2:**
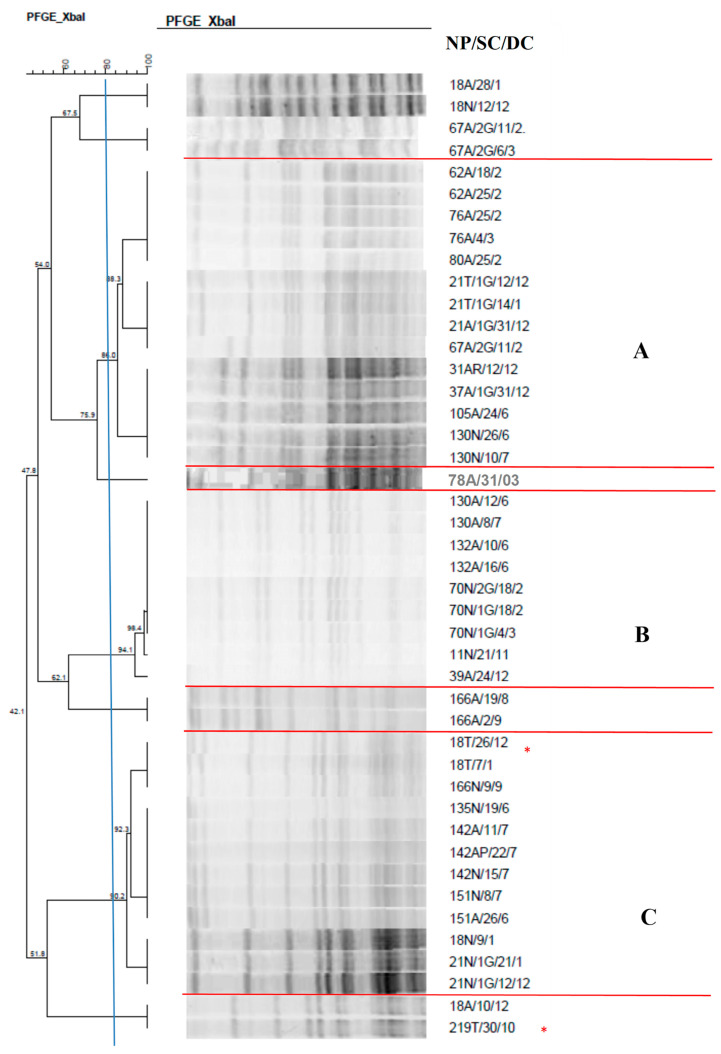
Dendrogram generated by Dice/UPGMA analysis (Bionumerics Applied Maths) [optimization: 1%, tolerance: 1.5%] of PFGE-XbaI profiles of *Serratia marcescens* isolates from newborns. Note: * isolate causing infection. NP: patient number; SC: collection site; DC: date of collection (day, month); A: anal; T: tracheal; G: twin. Letters A–C: Clusters, isolates with 80% or more similarity.

The dendrogram of the 44 *S. marcescens* isolates (Figure 2) revealed three clusters identified from A–C, with the clusters containing 9 to 14 isolates. Cluster A, made up of 14 isolates, showed a high rate of similarity between *S. marcescens* isolates from different sites and from different neonates; of these, 2 (14.3%) were isolated from nasal mucosa, 2 (14.3%) from tracheal aspirate, and the remaining from anal mucosa.

In cluster C, it was possible to observe the grouping of 12 isolates with a high similarity rate in different newborns, indicating the spread of this strain in the NICU. This cluster is noteworthy since one isolate from tracheal aspirate caused pneumonia in newborns, demonstrating the colonization and infection capacity of this microorganism.

It is worth mentioning that two isolates present in this dendrogram caused pneumonia in newborns.

**Figure 3:**
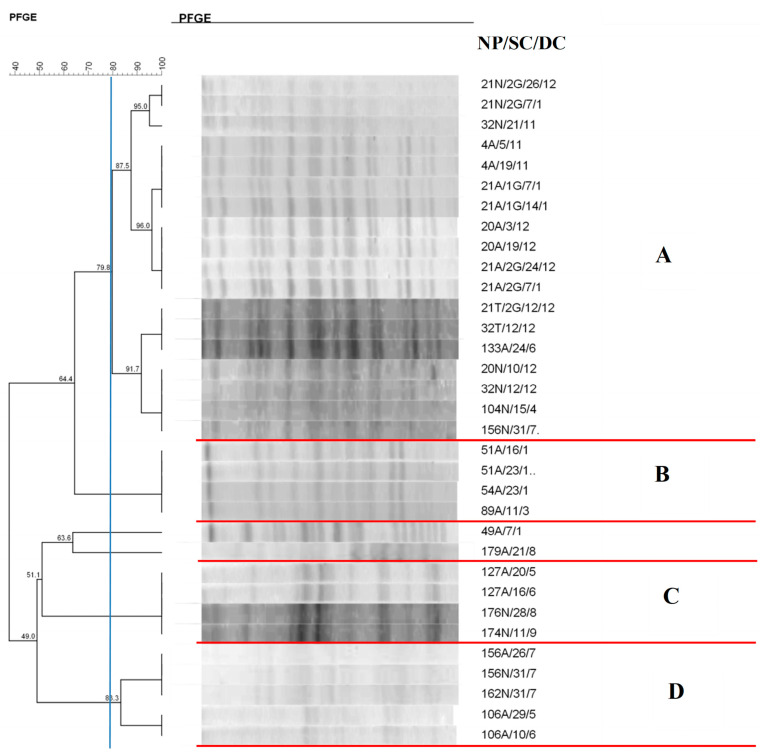
Dendrogram generated by Dice/UPGMA analysis (Bionumerics Applied Maths) [optimization: 1%, tolerance: 1.5%] of PFGE-XbaI profiles of *Escherichia coli* isolates from newborns. Note: SC: collection site; DC: date of collection (day, month); A: anal; T: tracheal; G: twin. Letters A–D: Clusters, isolates with 80% or more similarity.

Regarding *E. coli* isolates (Figure 3), four clusters were formed, with 18 isolates from different newborns in cluster A and several isolates showing 100% similarity.

In clusters B, C, D, and E, identical isolates colonizing the same newborn collected on different dates are observed.

**Figure 4:**
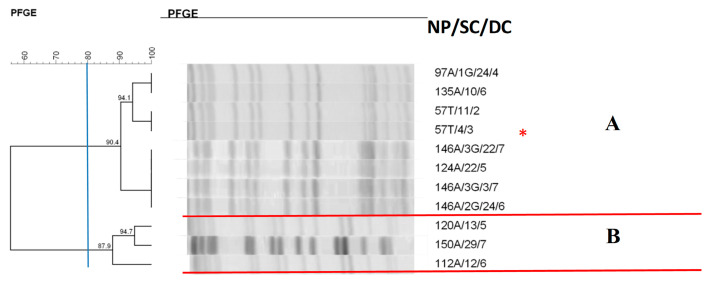
Dendrogram generated by Dice/UPGMA analysis (Bionumerics Applied Maths) [optimization: 1%, tolerance: 1.5%] of PFGE-XbaI profiles of *Klebsiella oxytoca* isolates from newborns. Note: * isolate causing infection; NP: patient number; SC: collection site; DC: date of collection (day, month); A: anal; T: tracheal; G: twin. Letters A–B: Clusters, isolates with 80% or more similarity.

The dendrogram of *K. oxytoca* isolates (Figure 4) showed the presence of two clusters; in cluster A, with eight isolates, colonization and subsequent infection by the same microorganism was observed. Cluster B contained isolates with a high similarity rate colonizing different newborns.

**Figure 5:**
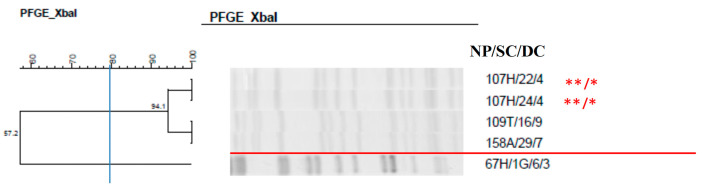
Dendrogram generated by Dice/UPGMA analysis (Bionumerics Applied Maths) [optimization: 1%, tolerance: 1.5%] of PFGE-XbaI profiles of *Klebsiella pneumoniae* isolates from newborns. Note: * isolates causing infection; ** ESBL-producing isolates confirmed by the disc approximation technique; NP: patient number; SC: collection site; DC: date of collection (day, month); A: anal; T: tracheal; G: twin.

The clonal profile of *K. pneumoniae* isolates (Figure 5) demonstrated the presence of a cluster with four isolates with a high similarity rate (94.1%); of these, two were obtained from blood cultures, one from anal mucosa, and one from tracheal aspirate (100% similarity) of different newborns; thus, this strain was present in the NICU colonizing or infecting different newborns, highlighting the production of ESBL by blood culture isolates.

**Figure 6:**
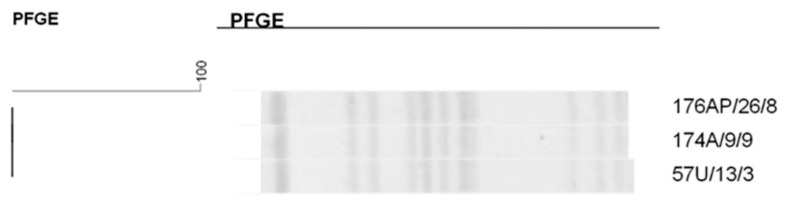
Dendrogram generated by Dice/UPGMA analysis (Bionumerics Applied Maths) [optimization: 1%, tolerance: 1.5%] of PFGE-XbaI profiles of *Proteus mirabilis* isolates from newborns. Note: NP: patient number; SC: collection site; DC: date of collection (day, month); A: anal; T: tracheal.

Analysis of the three *P. mirabilis* isolates (Figure 6) showed 100% similarity between isolates obtained from colonization and urinary infection of different newborns.

**Figure 7:**
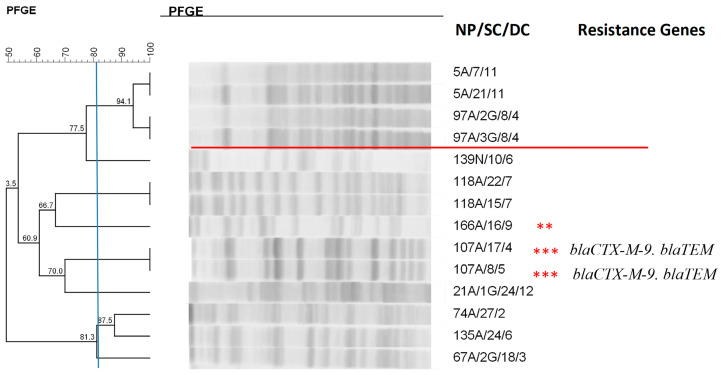
Dendrogram generated by Dice/UPGMA analysis (Bionumerics Applied Maths) [optimization: 1%, tolerance: 1.5%] of PFGE-XbaI profiles of *Enterobacter aerogenes* isolates from newborns. Note: ** ESBL-producing isolates confirmed by the disc approximation technique; *** ESBL-producing isolates that carried resistance genes; NP: patient number; SC: collection site; DC: date of collection (day, month); A: anal; N: nasal; G: twin.

Analysis of the clonal profile of the *E. aerogenes* isolates (Figure 7) revealed a cluster of four isolates from two newborns, showing 94.1% similarity.

**Figure 8:**
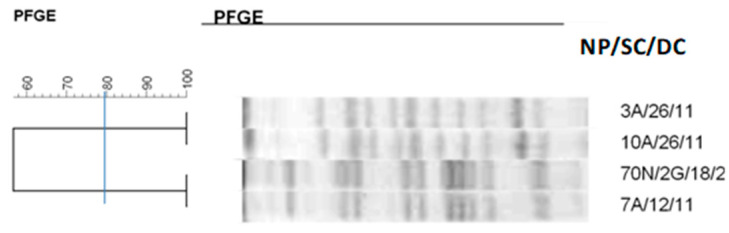
Dendrogram generated by Dice/UPGMA analysis (Bionumerics Applied Maths) [optimization: 1%, tolerance: 1.5%] of PFGE-XbaI profiles of *Citrobacter freundii* isolates from newborns. Note: NP: patient number; SC: collection site; DC: date of collection (day, month); A: anal; N: nasal; G: twin.

**Figure 9:**
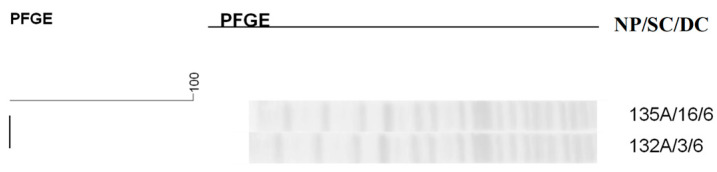
Dendrogram generated by Dice/UPGMA analysis (Bionumerics Applied Maths) [optimization: 1%, tolerance: 1.5%] of PFGE-XbaI profiles of *Citrobacter diversus* isolates from newborns. Note: NP: patient number; SC: collection site; DC: date of collection (day, month); A: anal; N: nasal; G: twin.

It was not possible to observe the formation of clusters in the dendrograms of *C. freundii* and *C. diversus* (Figure 8 and Figure 9), although some isolates showed high similarity, demonstrating greater genetic variability, with different strains isolated from newborns.

**Figure 10:**
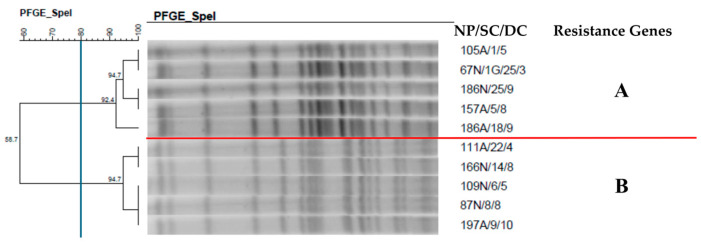
Dendrogram generated by Dice/UPGMA analysis (Bionumerics Applied Maths) [optimization: 1%, tolerance: 1.5%] of PFGE-Spel profiles of *Pseudomonas aeruginosa* isolates from newborns. Note: NP: patient number; SC: collection site; DC: date of collection (day, month); A: anal; N: nasal. Letters A–B: Clusters, isolates with 80% or more similarity.

The clonal profile of the *P. aeruginosa* isolates (Figure 10) demonstrated the presence of two clusters with five isolates from colonization of anal and nasal mucosa, showing a high similarity rate (92.4% and 94.7%). Different newborns colonized by the same pathogen were found in both clusters.

**Figure 11:**
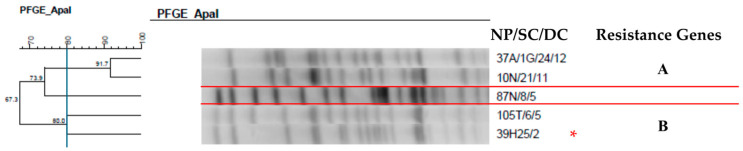
Dendrogram generated by Dice/UPGMA analysis (Bionumerics Applied Maths) [optimization: 1%, tolerance: 1.5%] of PFGE-Spel profiles of *Acinetobacter baumannii* isolates from newborns. Note: * isolate causing infection; NP: patient number; SC: collection site; DC: date of collection (day, month); A: anal; N: nasal; T: tracheal; H: blood culture. Letters A-B: Clusters, isolates with 80% or more similarity.

Analysis of the clonal profile of the A. baumannii isolates (Figure 11) revealed two clusters containing two isolates from different newborns. It is worth noting that in one of the clusters, the same pathogen was found in tracheal colonization and causing bloodstream infection (BSI) in newborns.

### 2.8. Analysis of Risk Factors

We also employed univariate and multivariate Cox regression analyses to identify risk factors for infection by Gram-negative microorganisms. The results of multivariate analysis revealed that the biofilm production ability of the microorganism increased by 75% (1.75 times) the daily probability of the newborn developing infection. Likewise, the production of ESBL by these pathogens increased the daily probability of infection by 46.8 times (Table 4).

## 3. Discussion

In view of the scarcity of data on the neonatal population, this study aimed to provide consistent and representative information on the genotypic and epidemiological characteristics of Gram-negative microorganisms isolated from colonization and infection sites in neonates admitted to a tertiary university center of high complexity.

During the study period, 51.9% of newborns were colonized and 3.9% were infected with Gram-negative microorganisms. Enterobacterales were the main microorganisms isolated from colonization and infection sites. This study demonstrated greater colonization of the anal mucosa, followed by the nasal mucosa.

Our results indicate that *E. cloacae* was the most frequently isolated bacterium in colonization samples. In the 1970s, Goldman et al. [16] observed that 52.0% of neonates were colonized by this microorganism. These results are compatible with those reported in 2019 by Baier et al. [17] and are also similar to those found in the present study (45.0%).

The gut could constitute a reservoir because *E. cloacae* establishes early in the neonatal microbiota. However, several outbreaks involving *E. cloacae* have been described over recent years, suggesting that the NICU environment or staff may also be a source of infection and transmission inside an NICU [18]. The fight against the establishment and transmission of *E. cloacae* and especially multidrug-resistant strains within a setting is a major challenge that involves respecting standard hygiene, reinforcing disinfection procedures, and restricting the use of broad-spectrum antibiotics [19]. 

The data found here revealed higher rates of infection by *K. pneumoniae* and *S. marcescens* that caused BSI and pneumonia, respectively. *K. pneumoniae* is known to cause nosocomial infections in premature neonates and several outbreaks have been reported over the past few years [20]. *S. marcescens* is an important nosocomial microorganism, especially in the NICU, which can cause serious infectious diseases including meningitis, bacteremia, and pneumonia [21]. In this study, high biofilm production was observed, with 95.0% of Enterobacterales and 100% of NFGNB being producers. A high proportion of Enterobacterales showed average biofilm production, while the majority of NFGNB were strong producers. A study conducted in four hospitals between 2016 and 2017 with isolates obtained from colonization and infection sites also revealed higher rates of biofilm production by *P. aeruginosa* when compared to Enterobacterales [22].

The investigation of genes encoding specific *P. aeruginosa* virulence factors showed that most of the isolates carried the genes for alginate, associated with bacterial adherence and biofilm production; hemolytic phospholipase C, related to the destruction of cell membranes and osmoprotectant function; exotoxin A, associated with tissue destruction and inhibition of the macrophage response; alkaline protease, related to tissue damage and inactivation of IgG; elastase, immunoglobulin degradation factor and complement factors and rhamnolipids, surfactant and associated with bacterial adhesion [23].

Regarding the virulence of *P. aeruginosa* isolates from newborns, another study conducted by our research group found similar results for *P. aeruginosa* isolates from patients with peritonitis undergoing peritoneal dialysis. The same virulence profile of *P. aeruginosa* has been reported in patients with fibrosectics [23] but differs from that of isolates obtained from urinary infections, water, and soil [24,25], which show a lower frequency of these virulence genes. A study carried out by the Tehran University of Medical Sciences with children undergoing intensive treatment also found a high frequency of *P. aeruginosa* isolates in urine samples that carried the *exoS* (92.95%), *lasB* (91.54%), and *plcH* (70.42%) genes [26].

As for the production of ESBL, the present study demonstrated that 5.3% of the isolated pathogens exhibited phenotypic production of these enzymes [27]. It is noteworthy that most of these microorganisms came from colonization sites and comprise species of *E. cloacae*, *E. aerogenes*, and two *K. pneumoniae* isolates that caused BSI. Evidence indicates that ESBL-producing *E. cloacae* and *K. pneumoniae* are frequently involved in NICU outbreaks, with emerging epidemiological potential [18,19,27].

ESBL-producing Enterobacterales are noteworthy for their rapid spread and the appearance of new variants [28]. Enzymes belonging to the CTX-M family are prevalent in South America, as well as in Spain and Eastern Europe [29]. In Brazil, variants of the CTX-M type predominate over enzymes of the TEM and SHV types, which are more prevalent in North America and Western Europe, respectively [30]. Our findings agree with these data, with six (5.3%) of the sixteen isolates that produced ESBL carrying resistance genes; of these, four (66.7%) carried the CTX-M-9 gene, three (50%) carried the TEM gene, and one (16.7%) was positive for the SHV and CMY-2 genes.

Therefore, in this study, the most frequent type of ESBL was CTX-M-9. A multicenter study carried out in the state of Rio de Janeiro revealed that these rates corresponded to 87%. This high frequency is related to the spread of plasmids encoding CTX-M located on mobile genetic elements [31].

The frequent isolation of ESBL-producing strains and the risk of therapeutic failure due to the administration of cephalosporins have led to a greater use of carbapenem antibiotics [32]. An important result of the present study is the absence of carbapenemase-producing isolates. The NICU in question uses antimicrobial therapy with carbapenems only in sporadic cases.

Analysis of the macro-restriction profile of the chromosomal DNA of Enterobacterales isolated from colonization and infection sites in newborns allowed us to identify the persistence and dissemination characteristics of some strains within the unit. The clonal profile of *E. cloacae* revealed the presence of 10 clusters that were named A-J. In all clusters, it was possible to observe the presence of different newborns harboring the same lineage of *E. cloacae* at different colonization sites. Cluster G grouped four isolates from the anal mucosa. One isolate produced ESBL in the phenotypic test and carried the TEM gene, while another isolate in the same cluster carried the CMY-2 and SHV genes. In contrast, in the same dendrogram, the presence of the *bla*CTX-M-9 gene was identified in two isolates that showed 100% similarity and that were obtained from different newborns. A study conducted with children from a Pediatric Emergency Center in Doha, Qatar, investigated the presence of phenotypic production of ESBL and resistance genes in enterobacteria isolated from urine samples. The data revealed a combination of two or more types of enzymes, including the *bla*CTX-M, *bla*TEM, and *bla*SHV genes [33]. Most of these isolates (59%) showed resistance mediated by the *bla*CTX-M gene. These findings are similar to the results of the present study that identified isolates carrying more than one resistance gene, with a predominance of CTX-M-9.

It is worth mentioning that in cluster C in which five *E. cloacae* strains isolated between February and October from different newborns were grouped, ESBL were phenotypically detected in three *E. cloacae* isolates that grouped with a high similarity rate; these isolates were found to be the same strain that possibly acquired this resistance during the NICU stay. A study investigating isolates of *Enterobacter* spp. obtained from different patients admitted to three teaching hospitals found that 27 of the 30 isolates exhibiting phenotypic production of ESBL carried the *bla*CTX-M, *bla*TEM, and *bla*SHV resistance genes [34]. In a survey conducted by Kanamori et al. [35] in Japan, 11 of 22 producer strains harbored these genes.

Analysis of the clonal profile of *S. marcescens* revealed the formation of three clusters, all demonstrating the cross-transmission of microorganisms. Cluster A comprised different newborns harboring identical isolates or isolates with a high similarity rate, which colonized and caused pneumonia. The strain associated with infection showed strong biofilm production. This pathogen is commonly found in hospitals where it causes infections. It presents high adherence capacity to medical devices due to biofilm production, favoring the development of infectious processes. A study carried out in an NICU in Mexico that investigated blood isolates, catheter tips, peritoneal fluid, pleural fluid, and abdominal abscesses demonstrated cross-transmission between NICU patients and other wards [36].

The clonal profile of *E. coli* revealed the formation of five clusters. The grouping of 12 isolates could be observed in cluster A. These isolates colonized different sites in the same neonate and in different newborns. Most of the strains showed 100% similarity. It is worth mentioning that over the 69-day period, this strain colonized different sites in five newborns, demonstrating its persistence during this period. Another important finding in this cluster is the same microorganism colonizing twin newborns. *E. coli* is usually a harmless commensal of the human gastrointestinal tract [37]. However, there are some highly virulent strains capable of causing intestinal and urinary tract infections, meningitis, intra-abdominal infection, pneumonia, infection of intravascular devices, osteomyelitis, and bacteremia [38,39]. As the main microorganism present in the maternal genital tract due to the proximity between the anal and perineal region, vaginal colonization by this microorganism can be detected in 7% to 13% of pregnant women [40,41].

Analysis of the *E. aerogenes* clonal profile demonstrated the formation of one cluster that contained four isolates from two different newborns, showing 94.1% similarity. Among the 14 isolates subjected to typing, 4 produced ESBL phenotypically; of these, 2 isolates from the same newborn collected on different dates carried the CTX-M-9 and TEM genes and showed 100% similarity. The presence of ESBL in this species represents a major problem due to the potential transmission of resistance genes to other bacterial species and because most ESBL are encoded by plasmids that also carry genes that confer resistance to other non-β-lactam antimicrobials such as aminoglycosides [42]. A study conducted with the pediatric population of Vara Hospital, located in western Bulgaria, revealed a predominance of the CTX-M enzyme of group 3 in *E. aerogenes* clones detected in two epidemics. This finding agrees with the data of the present study and other investigations that identified *E. aerogenes* clones carrying CTX-M enzymes. Ghanavati et al. [34] demonstrated that *bla*CTX-M and *bla*TEM were the most common resistance genes found in this species.

In the *K. oxytoca* isolates, the formation of two clusters (A and B), cluster A, was observed, showing isolates from anal colonization and isolated from the tracheal aspirate associated also with pneumonia. It is worth mentioning that the isolate causing infection exhibited strong biofilm production. *K. oxytoca* is an opportunistic pathogen that frequently causes pneumonia, bacteremia, urinary tract infections, and enterocolitis [43,44]. A study conducted in Japan analyzed the genetic profile of *K. oxytoca* involved in an outbreak lasting one year and six months and also detected identical isolates, indicating that the outbreak was caused by the same strain [45].

The genetic profile of *K. pneumoniae* demonstrated the presence of one cluster containing colonization and blood culture isolates associated with BSI. These isolates showed phenotypic production of ESBL and strong biofilm production. A study that investigated clinical samples collected from patients admitted to the NICU, Pediatric ICU, and Adult and Coronary ICU of a hospital in Southern Chile by analysis of the genetic profile also demonstrated a high rate of similarity between isolates; in addition, isolates producing ESBL phenotypically did not carry resistance genes [46]. One of the main virulence factors of *K. pneumoniae* includes the production of biofilm, favoring the process of adherence to medical devices and hindering the treatment of infectious processes through their physiological and genetic interactions [47].

Analysis of the chromosomal DNA macro-restriction profile of *P. mirabilis* isolates revealed 100% similarity between isolates obtained from anal colonization and urinary tract infection in different newborns. Isolates from colonization and infection sites showed average biofilm production. *P. mirabilis* causes symptomatic urinary tract infections, including cystitis and pyelonephritis, and is found in cases of asymptomatic bacteriuria. This bacterium harbors some virulence factors involved in this process, including the ability of biofilm production [48,49].

The clonal profile of the *P. aeruginosa* isolates demonstrated the presence of two clusters containing five isolates from colonization of anal and nasal mucosa, with a high similarity rate. Different newborns colonized by the same pathogen were found in both clusters. In addition, these strains persisted in the NICU, colonizing newborns during a large part of the study. *P. aeruginosa* is the causative agent of a wide range of infections in NICUs, including sepsis, pneumonia, meningitis, diarrhea, conjunctivitis, and skin infections [50]. Transmission of this pathogen is often associated with environmental reservoirs and the hands of healthcare professionals [50,51].

Despite the polyclonal profile of *A. baumannii*, two clusters containing two isolates from different newborns were observed. It is worth noting that in one of the groups, this species appeared in tracheal colonization and as the causative agent of BSI. Over the years, *A. baumannii* has become a leading cause of nosocomial outbreaks. The use of broad-spectrum antibiotics and invasive procedures such as endotracheal tubes, intravenous catheters, and urinary catheters favor colonization and infection by these pathogens [52,53].

We also employed univariate and multivariate Cox regression analyses to identify risk factors for infection by Gram-negative microorganisms. The results of multivariate analysis revealed that the biofilm production ability of the microorganism increased by 75% (1.75 times) the daily probability of the newborn developing infection. Biofilms are a known cause of a large percentage of nosocomial infections [54]. This increased risk of developing infection may be related to antimicrobial resistance [55] as a result of reduced penetration of the antibiotic through the biofilm matrix and a change in the growth rate of the pathogens composing the biofilm [56], as well as physiological alterations, including the expression of possible resistance genes [57]. Infections caused by microorganisms capable of producing biofilms are often difficult to eradicate and can evolve to recurrent infections [58,59]. This fact becomes an extremely serious problem, especially for the neonatal population that has a weakened immune system and is frequently exposed to various invasive procedures. Studies have compared the percentage of deaths caused by bacteria in suspension and biofilms and the results showed that biofilm producers exhibit a high rate of resistance to different antimicrobials [60,61].

Like biofilm production, in the present study, the production of ESBL increased the daily probability of the newborn developing infection by 46.8 times. HAIs caused by ESBL-producing microorganisms are a growing concern in NICUs and are frequently associated with hospital outbreaks [62]. Studies have shown that the length of hospital stay of newborns is often associated with the development of infections caused by ESBL-producing bacteria, as are immaturity of the immune system, the frequent use of invasive devices, and the environment [63,64]. Furthermore, the empirical use of antimicrobials can favor selective pressure and consequently increase the rates of colonization and infection by ESBL-producing microorganisms [65].

This study has some limitations, including the lack of access to isolates obtained in recent years. Further studies are therefore needed to evaluate changes in the antimicrobial resistance profile and mechanisms over time. Knowledge of resistance mechanisms, in conjunction with rapid laboratory diagnosis of multidrug-resistant strains, is essential for choosing the appropriate antibiotic therapy and consequently controlling the spread of bacterial resistance.

## 4. Materials and Methods

### 4.1. Study Location

The study was carried out using strains of Gram-negative bacteria isolated from newborns admitted to the NICU of HFMB, Botucatu, Brazil, a tertiary university center of high complexity. The unit currently has 15 beds and is a regional reference for the care of patients of the Unified Health System (UHS) from the southwestern region of the State of São Paulo and other neighboring states.

Inclusion criteria: Gram-negative bacteria previously collected in a prospective cohort study conducted from November 2013 to November 2014 from tracheal aspirate, nasal mucosa, and anal mucosa samples obtained from NICU newborns of HFMB, Botucatu, Brazil.

Exclusion criteria: Other microorganisms collected in a prospective cohort study conducted from November 2013 to November 2014 from tracheal aspirate, nasal mucosa, and anal mucosa samples obtained from NICU newborns of HFMB, Botucatu, Brazil.

Definitions:

Colonization: Presence of the microorganism in the host in the absence of clinical manifestations and an immune response at the time of bacterial isolation [66].

Infection/HAI: Presence of clinical signs of infection (such as temperature instability, cardiorespiratory or gastrointestinal disturbances, lethargy, irritability) along with laboratory evidence (abnormal white blood cell count and elevated acute phase reactants) occurring 72 h after birth and treated with appropriate antibiotics [67].

One of the following criteria was used for the diagnosis of BSI:

Criterion 1: One or more blood cultures positive for microorganisms that do not contaminate the skin and the microorganism is not related to infection elsewhere.

Criterion 2: At least one of the following signs and symptoms in the absence of any other recognized non-infectious cause and unrelated to infection at another site (discussion with the newborn’s attending physician): thermal instability, bradycardia, apnea, food intolerance, worsening of respiratory distress, glucose intolerance, hemodynamic instability, hypoactivity/lethargy.

- Common skin contaminating microorganisms: *Corynebacterium* spp. (excluding *C. diphtheriae*), *Bacillus* spp. (excluding *B. anthracis*), *Propionibacterium* spp., coagulase-negative *Staphylococcus*, *Streptococcus* of the viridans group, *Aerococcus* spp., and *Micrococcus* spp., isolated from at least two blood cultures collected at two different sites, with a maximum interval of 48 h between samplings.
- Coagulase-negative *Staphylococcus* isolated from at least one peripheral blood culture of a patient with a central vascular catheter.

### 4.2. Collection and Genotypic Identification of Microorganisms

The strains were previously collected from nasal mucosa, anal mucosa, and tracheal aspirate samples in a prospective cohort study conducted from November 2013 to November 2014 from neonates born and admitted to the NICU of HFMB, twice a week during the newborn’s stay in the NICU. During the study period, 600 positive colonization samples showing growth of microorganisms were obtained. Of these, 350 isolates exhibited specific morphology and staining for Gram-negative bacilli and underwent genotypic identification. After DNA extraction with the Illustra Kit (GE Healthcare, Little Chalfont, Buckinghamshire, UK), Gram-negative bacilli were identified by PCR using conserved primer sequences adjacent to the 16S genes, specific to each species [68,69,70,71,72,73]. Bacteria that could not be identified by PCR were submitted to identification by matrix-assisted laser desorption ionization-time of flight mass spectrometry (MALDI-TOF MS) in the VITEK MS system (bioMérieux, Durham, NC, USA) (Table 1 and Table 2).

During the study, the newborns were followed up until the final outcome of hospital discharge or death; in the event of infection during the hospitalization period, the clinical materials obtained for diagnosis at the Clinical Laboratory of HFMB were also included in the study.

### 4.3. Biofilm Production

To evaluate the capacity of the isolates to adhere to abiotic surfaces, polystyrene plates with 96 holes were used as previously described [74]. The score used to classify biofilm production was divided into four categories: non-producing strain (when the OD of the strain was lower than the OD of the blank), weak (OD of the strain between the OD of the blank and 2× the OD of the blank), medium (OD of the strain between 2× the OD of the blank and 4× the OD of the blank), and strong (OD of the strain greater than 4× the OD of the blank) [75].

### 4.4. Confirmation of the Production of Extended-Spectrum β-Lactamases

The production of ESBL was confirmed by the disc approximation technique. An amoxicillin disc with clavulanic acid was placed in the center of the plate and 30 mm (from center to center) away from the other β-lactam discs: ceftazidime, cefotaxime/ceftriaxone, and aztreonam [64,76]. This technique is not performed for NFGNB because of their intrinsic resistance to most β-lactams [77].

### 4.5. Molecular Characterization of Strains Producing Extended Spectrum β-Lactamases

All ESBL-producing strains identified by the phenotypic methods were evaluated by PCR for the detection of genes related to these phenotypes [24,78,79,80,81].

### 4.6. Evaluation of the Virulence Profile of Pseudomonas aeruginosa Isolates

The virulence profile of the isolates was only evaluated by PCR, in which the presence of the following genes was determined as described by Lanotte et al. [24]: alginate (*algD*), elastase (*lasB*), hemolytic phospholipase C (*plcH*), non-hemolytic phospholipase C (*plcN*), exoenzyme S (*exoS*), and exotoxin A (*toxA*).

### 4.7. Pulsed-Field Gel Electrophoresis (PFGE)

PFGE of colonizing microorganisms (swabs) and isolates from clinical materials was performed according to the protocol recommended by PulseNet [82]. Enzymatic restriction was carried out using *XbaI*. Electrophoresis was performed in a CHEF-DR III System (BioRad Laboratories, Hercules, CA, USA). Lambda PFG Ladder (New England BioLabs, Ipswich, MA, USA) was used as a molecular marker.

Similarity analysis was performed using the BioNumerics software (version 7.6; Applied Maths, Kortrijk, Belgium). The dendrograms were created using the UPGMA method (Unweighted Pair Group Method with Arithmetic Mean), with band position tolerance and optimization adjusted to 1.5% and 1%, respectively. A Dice similarity coefficient ≥ 80% was chosen to determine the clusters. Groupings with three or more isolates with similarity ≥ 80% were defined as a cluster.

### 4.8. Statistical Analysis

Data were analyzed using the SPSS 20.0 software (IBM, Armonk, NY, USA). Univariate and multivariate Cox regression analyses were performed to identify risk factors associated with colonization of newborns by Enterobacterales and/or NFGNB. The forward selection strategy was used to select variables for inclusion in multivariate analysis, with the variables being included sequentially in the model according to increasing *p*-value. Variables with *p* < 0.05 remained in the model.

## 5. Conclusions

Enterobacterales were the main microorganisms isolated from colonization and infection sites. Our results indicate that *Enterobacter cloacae* was the most frequently isolated bacterium in colonization samples. The data found here revealed higher rates of infection by *Klebsiella pneumoniae* and *Serratia marcescens* that caused bloodstream infection and pneumonia, respectively.

Enterobacterales showed possible cross-transmission and average biofilm production, while the majority of NFGNB were strong producers. The present data increase our knowledge of the molecular epidemiology of important Enterobacterales species, with emphasis on ESBL-producing *E. cloacae* and *K. pneumoniae* with emerging epidemiological potential in the NICU of a tertiary university hospital. Furthermore, the results highlight the need for the monitoring and implementation of control measures and for restricting the use of broad-spectrum antibiotics. 

## Figures and Tables

**Table 1:** Enterobacterales isolated from the nasal mucosa, anal mucosa and tracheal aspirate of neonates in the Neonatal Intensive Care Unit of the Botucatu Medical School, Botucatu, Brazil.

	Total Number of Isolates		Nasal Mucosa		Anal Mucosa		Tracheal Aspirate	
	N	(%)	N	(%)	N	(%)	N	(%)
*Enterobacter cloacae*	135	(45)	30	(22.2)	101	(74.9)	4	(2.9)
*Serratia marcescens*	73	(24.3)	16 ***	(21.9)	53 ****	(72.6)	4 **	(5.5)
*Escherichia coli*	45	(15)	14	(31.1)	29	(64.4)	2	(4.5)
Enterobacter aerogenes *	17	(5.7)	1	(5.9)	16	(94.1)	-	-
Klebsiella oxytoca *	12	(4)	-	-	10 *^/^	(83.3)	2 *^+^	(16.7)
Citrobacter freundii *	7	(2.3)	-	-	7	(100)	-	-
*Klebsiella pneumoniae*	4	(1.3)	-	-	2 *^|^	(50)	2 *^|^	(50)
Citrobacter diversus *	2	(0.7)	-	-	2	(100)	-	-
*Proteus mirabilis*	2	(0.7)	-	-	2 *^^^	(100)	-	-
Raoultella planticola *	1	(0.3)	-	-	1	(100)	-	-
Raoultella ornithinolytica *	1	(0.3)	-	-	1	(100)	-	-
Cedecea neteri *	1	(0.3)	-	-	1	(100)	-	-
Total	300	(100)	61	(20.3)	225	(75)	14	(4.7)

N: Number of isolates. * Microorganisms identified by matrix-assisted laser desorption ionization-time of flight (MALDI-TOF) mass spectrometry in the VITEK MS system (bioMérieux); ** three isolates associated with pneumonia; *** seven isolates associated with pneumonia; **** four isolates associated with pneumonia; *^/^six isolates associated with bloodstream infection; *^+^ two isolates associated with bloodstream infection; *^|^ one isolate associated with bloodstream infection; *^^^ two isolates associated with urinary tract infection.

**Table 2:** Non-fermenting Gram-negative bacilli isolated from the nasal mucosa, anal mucosa and tracheal aspirate of neonates in the Neonatal Intensive Care Unit of the Botucatu Medical School, Botucatu, Brazil.

	Total Number of Isolates		Nasal Mucosa		Anal Mucosa		Tracheal Aspirate	
	N	%	N	%	N	%	N	%
*Pseudomonas aeruginosa*	38	(76)	16	(42.1)	22	(57.9)	-	-
*Acinetobacter baumannii*	10	(20)	2 ***	(20)	7 **	(70)	1 **	(10)
Acinetobacter lwoffii *	2	(4)	-	-	2	(100)	-	-
Total	50	(100.0)	18	(36.0)	31	(62)	1	(2.0)

N: Number of isolates. * Microorganisms identified by matrix-assisted laser desorption ionization-time of flight (MALDI-TOF) mass spectrometry in the VITEK MS system (bioMérieux); ** one isolate associated with bloodstream infection; *** two isolates associated with bloodstream infection.

**Table 3:** Resistance genes detected in Enterobacterales isolated from the nasal mucosa, anal mucosa, and blood culture of neonates in the Neonatal Intensive Care Unit of the Botucatu Medical School, Botucatu, Brazil.

Isolate	Species	Resistance Genes
21A/1G/24/12	*Enterobacter aerogenes*	-
107A/17/4	*Enterobacter aerogenes*	*blaCTX-M-9*, *blaTEM*
107A/8/5	*Enterobacter aerogenes*	*blaCTX-M-9*, *blaTEM*
166A/16/9	*Enterobacter aerogenes*	-
78A/11/3	*Enterobacter cloacae*	-
78A/20/3	*Enterobacter cloacae*	-
69A/4/3	*Enterobacter cloacae*	*blaCTX-M-9*
107A/24/4	*Enterobacter cloacae*	*blaCMY-2*, *blaSHV*
107A/15/5	*Enterobacter cloacae*	*blaTEM*
167A/14/8	*Enterobacter cloacae*	*blaCTX-M-9*
182A/7/10	*Enterobacter cloacae*	-
44N/16/1	*Enterobacter cloacae*	-
147N/31/7	*Enterobacter cloacae*	-
166N/19/8	*Enterobacter cloacae*	-
107H/22/4	*Klebsiella pneumoniae*	-
107H/24/4	*Klebsiella pneumoniae*	-

G: twin newborn; N: nasal mucosa; A: anal mucosa; H: blood culture.

**Table 4:** Univariate and multivariate analysis (Cox regression) of predictors of infection by Enterobacterales and non-fermenting Gram-negative bacilli among neonates in the Neonatal Intensive Care Unit of the Botucatu Medical School, Botucatu, Brazil.

Predictor	Univariate Analysis		Multivariate Analysis	
	HR (95%CI)	*p*	HR (95%CI)	*p*
Male sex	0.59 (0.10–3.43)	0.56		
Birth weight (g × 100)	0.81 (0.61–1.02)	0.16		
Cesarean delivery	0.94 (0.16–5.63)	0.95		
Gestational age (weeks)	0.80 (0.59–1.09)	0.16		
Umbilical catheter	4.79 (0.55–42.06)	0.16		
Peripherally inserted central catheter	1.81 (0.21–15.84)	0.59		
Intravenous catheter	0.04 (0.00–...)	0.64		
Peripheral venous access	0.20 (0.04–1.13)	0.07		
Parenteral nutrition	33.74 (0.01–...)	0.38		
Surgery	0.59 (0.07–5.25)	0.64		
Mechanical ventilation	20.06 (0.00–...)	0.54		
Use of antimicrobials in the first 72 h	4.99 (0.58–43.39)	0.15		
Biofilm	1.69 (0.99–2.90)	0.053	**1.75 (1.10–3.04)**	**0.048**
ESBL	42.10 (3.80–463.13)	0.002	**46.79 (4.18–523.72)**	**0.002**

HR: hazard ratio; 95%CI: 95% confidence interval; ESBL: extended-spectrum β-lactamases.

## Data Availability

The data presented in this study are original and have not been published in scientific journals. The only document that contains these data is the doctoral thesis of Thaís Alves Barbosa, openly available in the Institutional Repository of UNESP at [http://hdl.handle.net/11449/194511] (accessed on 22 November 2024).
